# Eastern Ohio Dental Association

**Published:** 1874-04

**Authors:** W. P. Rice

**Affiliations:** Sec’y Pro Tem.


### EASTERN OHIO DENTAL ASSOCIATION.

The sixth semi-annual meeting of the Eastern Ohio Dental Association was held at the office of Dr. J. W. Lyder. in Alliance. Ohio, on Tuesday, Febuary 3d, 1874, Dr. J. H. Siddall, President, in the chair.

The Secretary being absent, Dr. W. P. Rice was appointed Secretary, *pro tem.* On account of the absence of the Secretary, the reading of the minutes of last meeting was dispensed with.

The following dentists were present, viz: Drs. Siddall, and Joseph Craig, Canton; Chas. Coffee and J. W. Lyder, Alliance; B. F. Gibbons, Youngstown; D. Gibbons, Warren; J. T. Barclay, Columbiana; W. P. Rice, Mt. Union; Jos. McMillen, East Fairfield; Dr. Swayne, Ravenna; J. C. Whinery, Salem.

On motion of Dr. Lyder, it was resolved that the first business of the Association be, to hear and take action on the report of the Committee appointed by the Ohio State Dental Society to solicit donations to aid in defence of the legal rights of the profession.

The report was made by Drs. Whinery and Lyder as said Committee. On motion, the report was received. Communications were read and received from Drs. Whitslar, Youngstown; Taylor, of East Fairfield; and McLean, of New Lisbon, all of which gentlemen promise and pledge themselves to abide by the action and decision of this Association. The question was discussed by Drs. D. Gibbons, B. F. Gibbons, J. C. Whinery, J. W. Lyder, C. P. Coffee, J. H. Siddall and others.

The following resolution, offered by Dr. Lyder, was unanimously adopted, viz: That we, as members of the Eastern Ohio Dental Associatien, heartily concur in the action adopted by the Ohio State Dental Society, in regard to the Cummings Patent.

An essay was read by Dr. Lyder on the subject of Dental Ethics. Dr. Siddall spoke of the open violation of the law regulating the practice of dentistry in this State by some dentists, and suggested the propriety of instituting legal proceedings against those who so violate it.

Dr. D. Gibbons is opposed to legal suasion and insists rather on educating the public so as to discriminate between the empiricism practiced by so-called dentists, or rather quacks, who are traveling over the country, soliciting patronage and inflicting upon the unsuspecting public their worthless dental operations; and those honorable and worthy dentists whose efforts to benefit the public and elevate the standard of the profession have and do insure and secure to them an office practice, both respectable and remunerative.

Dr. Barclay spoke of the impossibility of educating and elevating this class of illiterate men who are engaged in the itinerate practice of dentistry,—thinks they lack the requisite moral qualifications essential to the high standard of our profession; and also disapproves of dentists taking boys as students. He thinks none but men, and they morally, intellectually and physically well developed, are suitable to fill the ranks of our profession.

Dr. J. C. Whinery spoke of the duty of dentists as to their honesty of purpose, and their doing to and with their fellow practitioners as they would have others do to them. He is opposed to soliciting business or work,—has no sympathy for itinerants: compares them to tinkers going with their pack of tools from house to house, begging for work; thinks it will be very difficult to elevate the standard of our profession, with an ignorant, unintelligent set of dentists; favors education and fellowship rather than compulsion by law; thinks the State Law has stimulated to higher attainments, and caused men to read and post up and take advance steps in the profession.

A general quiz was held and conducted by the President.

*Ques.*—Have you ever made a nerve filling in an inferior incisor?

Dr. D. Gibbons has.

*Ques.*—What has been the success in capping nerves?

Dr. Whinery frequently succeeds, yet not always, as it is sometimes attended by congestion.

Dr. B. F. Gibbons attributes his former failures to lack of thorough excavating. Removes all and everything foreign or in any way diseased, even if severe pain and bleeding ensue; then saturate oxide of zinc with carbolic acid and apply to the point of exposure of nerve; then cap with chloride of zinc and fill permanently.

Dr. D. Gibbons excavates very thoroughly and caps almost exclusively with cilicious cements, where health, age and condition of patient are favorable; expects favorable results.

*Ques.*—What is the cause of the pain produced in the tooth with a metalic filling upon contact with other metalic fillings?

*Ans.*—It is caused by galvanic action, owing to the contact of the two different metals in the acid fluids of the mouth. Pain is also frequently caused by thermal changes, as where fluids either hot or cold are brought in contact with large metalic fillings.

Several members of the Association gave their experience and practice in the treatment of abscessed inferior incisors, which in the main is the same as the treatment for any other tooth similarly affected.

Dr. Barclay has bleached teeth with oxy-chloride of zinc, with very good effect; lets considerable of the oxy-chloride remain under the gold filling.

Dr. Siddall has succeeded well by applying nitric acid, perhaps one minute, then cleanse thoroughly with soda and fill.

Dr Lyder thinks the discoloration following the application of nitric acid is caused by not excavating deeply and thoroughly; that the acid follows and penetrates the sensitive dentine and leaves its outline on the remaining tooth substance.

Dr. B. F. Gibbons believes in the theory of the open-mouthed dentinal tubes, or tubuli, absorbing the disintegrated matter, thus causing the discoloration.

*Ques.*—When a patient presents and demands the extraction of good, sound teeth or those that could be filled and rendered serviceable, shall a dentist extract such teeth and be justified in so doing?

In the discussion of this question the opinion seemed to prevail that an honest, conscientious dentist could not with propriety indiscriminately sacrifice such teeth, to please his patients, without compromising his principles.

*Ques.*—Does a tooth grow up or down, as to its elongation?

Dr Whinery said that he has had considerable experience in viewing the development of teeth, by observing comparative anatomy in animals, and has observed the development of the roots from time to time by the addition of the earthy matter and lessening of the vital tissues. The ossification in a molar tooth is from three or four points.

The relative properties of creosote and carbolic acid as therapeutical agents were fully discussed.

MISCELLANEOUS BUSINESS BEING IN ORDER.

On motion, Drs. Lyder and Whinery were elected delegates to represent this Association at the American Dental Association, to be held in Detroit, Aug. 4th, 1874.

The President appointed Drs. D. Gibbons, J. W. Lyder and J. C. Whinery, as Examining Committee; and Drs. C. P. Coffee, B. F. Gibbons and J. T. Barclay, Executive Committee.

The Treasurer reported $14.05 in the treasury.

A bill presented by J. M. Porter was, on motion, ordered paid.

The President appointed as Essayists: Dr. Whinery, subject, Dentistry Thirty Years ago; Dr. Barclay, to make his own selection of subject.

Dr. Lyder was appointed to give a clinic by preparing and filling an inferior molar on the posterior surface.

It was moved by Dr. Whinery that the acting Secretary of last meeting be requested to furnish the Secretary of this meeting with the minutes and proceedings of the last Association, for publication.

On motion, adjourned to meet on the first Tuesday of February next.

W. P. Rice,
*Sec'y pro tem.*

